# Real‐world genomic landscape of colon and rectal cancer

**DOI:** 10.1002/2211-5463.13957

**Published:** 2025-01-26

**Authors:** Markus Schulze, XiaoZhe Wang, Jawad Hamad, Julia C. F. Quintanilha, Lincoln W. Pasquina, Julia F. Hopkins, Juergen Scheuenpflug, Zheng Feng

**Affiliations:** ^1^ Clinical Measurement Sciences, Global Research & Development Merck KGaA Darmstadt Germany; ^2^ Clinical Measurement Sciences, Global Research & Development EMD Serono Billerica MA USA; ^3^ Medical Unit Oncology Merck Healthcare KGaA Darmstadt Germany; ^4^ Foundation Medicine Inc Cambridge MA USA

**Keywords:** *BRAF*, colorectal cancer, *RAS*, real‐world data

## Abstract

*MAPK* signaling activation is an important driver event in colorectal cancer (CRC) tumorigenesis that informs therapy selection, but detection by liquid biopsy can be challenging. We analyze real‐world comprehensive genomic profiling (CGP) data to explore the landscape of alterations in *BRAF* or *RAS* in CRC patients (*N* = 51 982) and co‐occurrence with other biomarkers. A pathogenic *RAS* or *BRAF* alteration was found in 63.2% and 57.9% of colon and rectal cancer samples, respectively. In a subset of 140 patients with both tissue‐ and liquid‐based CGP, the sensitivity of liquid for results found by tissue was 100% when ctDNA tumor fraction was at least 1%, illustrating the utility of tissue and liquid biopsy in detecting driver alterations in CRC.

## Graphical abstract

We confirmed enrichment of RTK alterations in patients with *RAS*/*BRAF*‐wt tumors and of *PI3K* pathway alterations in *RAS*/*BRAF*‐altered tumors with 51 982 real‐world tissue comprehensive genomic profiling (CGP) samples in CRC. In a subset of 140 patients with both tissue and liquid results, the sensitivity of liquid for results found by tissue was 100% when ctDNA tumor fraction was at least 1%.

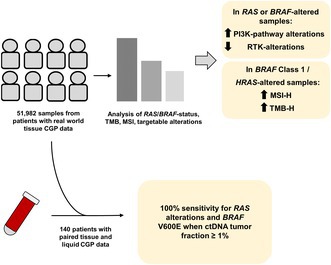

**Abbreviations**
*BRAF*
: B‐Raf proto‐oncogene, serine/threonine kinase
CGP: comprehensive genomic profiling
CN: copy number event
CRC: colorectal cancer
ctDNA: circulating tumor DNA
Indel: insertion/deletion
*KRAS*
: *KRAS* proto‐oncogene, GTPase
*MAPK*
: mitogen‐activated protein kinase
MSI‐H: microsatellite instability high
MSI‐L: microsatellite instability low
MSS: microsatellite stable
mut/MB: mutations per megabase
NPV: negative predictive value
*NRAS*
: NRAS proto‐oncogene, GTPase
PPA: positive percent agreement
RE: rearrangement
RWD: real‐world data
SNV: single‐nucleotide variant
TF: tumor fraction
TMB‐H: tumor mutational burden high
TMB‐L: tumor mutational burden low
wt: wild‐type

Molecular profiling of colorectal cancer (CRC), the third most common cancer worldwide [1], has revealed that activation of *WNT*, *MAPK*, and *PI3K* signaling as well as alterations in TGF‐beta and DNA damage response pathways are common features of CRC [2, 3].

*MAPK* signaling is frequently activated by alterations in *KRAS*, *NRAS*, or *BRAF* in CRC [4]. The *RAS* family consists of three closely related genes—*KRAS*, *NRAS*, and *HRAS*—with *KRAS* being the most commonly altered *RAS* family gene in CRC, followed by *NRAS*, whereas *HRAS* alteration is a very rare event [5, 6]. *KRAS* or *NRAS* alterations are important driver events in CRC tumorigenesis, a known predictive marker for targeted therapies and critical for treatment selection [7].

*BRAF* mutations can be subclassified into three distinct classes: Class 1 mutants that occur at position V600 signal as constitutively active monomers and are the most common class in CRC; class 2 mutants that form RAS‐independent RAF dimers; and class 3 mutants that have low or no kinase activity but act by amplifying upstream signals in the *MAPK* pathway [8].

Importantly, *RAS* and *BRAF* alterations are largely mutually exclusive [9], albeit class 3 mutations have been described to co‐occur more frequently with *RAS* alterations [3].

Class 1 mutants, on the other hand, are highly overrepresented in colorectal cancer with high microsatellite instability [7].

Tumors that do not harbor *RAS*/*BRAF* mutations show an overrepresentation of alterations that also induce activation of the *MAPK* pathway, *for example*, activating alterations in RTKs [10, 11].

Such alterations have been described in many different RTKs in CRC. Fusions of RTKs, *that is*, *ALK*, *ROS1*, *RET*, and *NTRK1‐3* that occur at low prevalence in CRC are prominent examples. Other activating genetic events can affect *EGFR*, *ERBB2*, or *FGFRs* [12].

First‐line targeted therapy in combination with chemotherapy is the standard of care for most metastatic CRC patients with the type of targeted agent (*e.g*., anti‐EGFR, anti‐VEGF) depending on tumor location and molecular status (microsatellite instability, *RAS*, and *BRAF* status) [13].

Advances have been made to target the *MAPK* pathway in the clinic, *for example*, in *KRAS* G12C or *BRAF* V600‐mutated cancers [12]. *KRAS* G12V and G12D inhibitors are also being developed [14].

However, the optimal treatment combination strategies across multiple lines of treatment still remain unclear.

Therefore, comprehensive clinical trial design is needed to accommodate for the complex and evolving genomic landscape and to enable precision medicine in clinical trials. Real‐world data (RWD) is an important tool to achieve this goal, as it enables deep analysis of large cohorts of patients with a diverse treatment history [15, 16]. Here, analysis of RWD clinical genomics was conducted using the FoundationInsights™ web platform to explore the landscape of *RAS*, *BRAF*, and co‐occurring alterations in CRC.

## Material and methods

The prevalence of gene alterations and their co‐occurrence with *RAS*/*BRAF* alterations in tumor tissue of CRC patients (*N* = 51 982) were investigated using the FoundationInsights™ web platform, which includes harmonized results from the FoundationOne® CDx [17], FoundationOne® Heme [18], and PD‐L1 assays from 2012 to March 2022. Data were collected from Foundation Medicine (FMI) solid (DNA‐based) and heme (DNA and RNA‐based) tests from 2012 to March 2022. Both assays are hybrid capture‐based next‐generation sequencing assays performed in a CLIA‐certified, CAP‐accredited laboratory (Foundation Medicine, Cambridge, MA, USA).

Where indicated, the patient samples were subdivided into colon cancer (*N* = 42 800) and rectal cancer (*N* = 9182) in the FoundationInsights™ web platform. Patient age was restricted from age 18–89.

Alterations included single‐nucleotide variants (SNVs) or insertions/deletions (Indels), rearrangements (RE), and copy number events (CN). Tumor mutational burden (TMB) and microsatellite stability (MSI) status were also characterized as previously described [19]. Samples with ≥10 mutations/megabase were considered as TMB‐high (TMB‐H).

Briefly, MSI stability status repetitive loci were assessed for length of repeats and compared to an internal database to determine locus stability. A sample with an increased fraction of unstable loci is considered to have high or low microsatellite instability (MSI‐H, MSI‐L) or were designated microsatellite stable (MSS).

PD‐L1 22C3 was run according to manufacturer instructions in a CLIA‐certified and CAP‐accredited laboratory (Foundation Medicine, Inc) and scored with a tumor proportion score = # PD‐L1‐positive tumor cells/(total # of PD‐L1‐positive + PD‐L1‐negative tumor cells). All patient cases were tested with manufacturer‐recommended system level controls, H&E‐stained slide, negative reagent control slide, and PD‐L1 22C3 IHC slide. CNAs were determined using a comparative genomic hybridization‐like method as described [19]. Briefly, a genome‐wide log‐ratio profile of the sample is obtained, then segmented, and interpreted using allele frequencies of sequenced single‐nucleotide polymorphisms to estimate tumor purity and copy number at each segment.

Statistical analyses were performed with R v4.2.1 and custom scripts and the R‐packages data.table 1.14.6, stringr 1.5.0, gsubfn 0.7, and dplyr 1.0.10.

*BRAF* mutations (SNVs or Indels) observed in our cohort were assigned to class 1, 2, or 3 based on available literature [8, 20, 21, 22, 23, 24]. We did not include *BRAF* fusions in this analysis, albeit *BRAF* fusions containing the kinase domain are class 2 variants.

To evaluate concordance between tissue and liquid biopsy‐based profiling, patients with a confirmed diagnosis of colorectal cancer who underwent both tissue (FoundationOne CDx) and liquid (FoundationOne® Liquid CDx) CGP testing within an interval of up to 90 days between tissue and liquid collection were included. Positive percent agreement (PPA/sensitivity) and negative predictive value (NPV) for *KRAS*, *NRAS*, and *BRAF* V600E mutation detection were calculated using the tissue test as truth for comparison. PPA and NPV were calculated for all patients with tissue and liquid samples and for those patients with liquid sample with ctDNA tumor fraction (TF) ≥1% [25, 26, 27, 28, 29]. ctDNA TF calculation was based on a composite algorithm incorporating multiple factors including aneuploidy, variant allele frequency, and canonical alterations. Briefly, when significant aneuploidy is present, a copy number model is constructed based on a panel of >30 000 commonly heterozygous single‐nucleotide polymorphisms across the genome. When significant aneuploidy is not detectable, ctDNA TF is determined based on variant allele frequencies of short variants deemed likely to be of somatic origin as informed by fragmentomic information [28]. Bar plots for specific *KRAS* and *NRAS* mutations were generated using R software version 2023.03.0 + 386.

Approval for this study, including a waiver of informed consent and a HIPAA waiver of authorization, was obtained from the Western Institutional Review Board (Protocol No. 20152817).

## Results

### *RAS/BRAF*‐alteration landscape in CRC

**Fig. 1:**
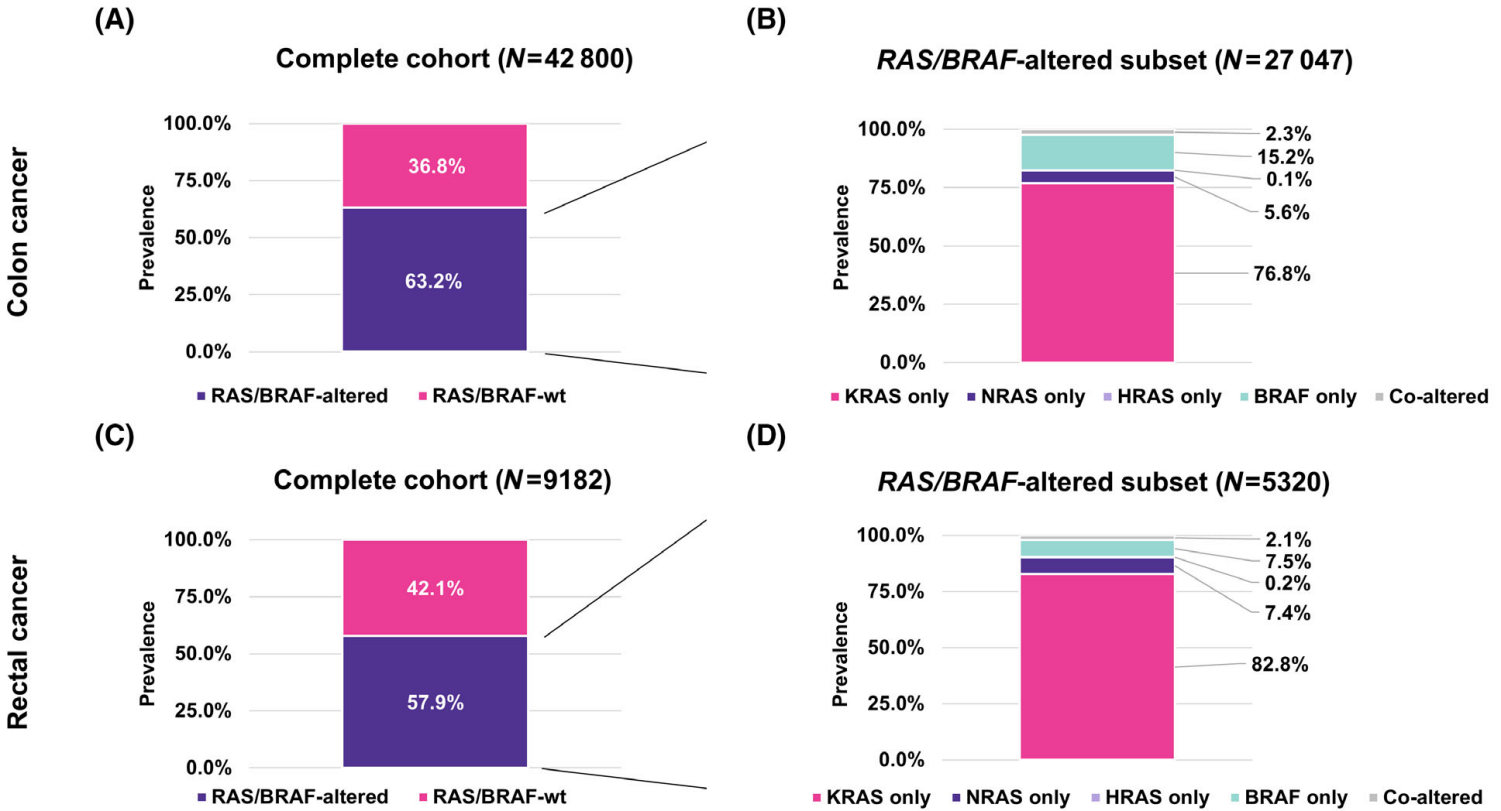
Prevalence and pattern of occurrence of *RAS/BRAF* alterations in colon and rectal cancer. (A) Percentage of *RAS/BRAF*‐altered (single‐nucleotide variant (SNV), insertion/deletion (Indel), copy number event (CN), or rearrangement (RE)) samples in the colon cancer cohort. Of all colon cancer samples (*N* = 42 800), 36.8% were *RAS*/*BRAF*‐wild‐type (wt) and 63.2% had a known or likely pathogenic *RAS*/*BRAF* alteration. (B) Percentage of *KRAS*, *NRAS*, *HRAS*, or *BRAF* alterations and their pattern of occurrence in the *RAS/BRAF*‐altered colon cancer cohort (*N* = 27 047). *KRAS*, *NRAS*, *HRAS*, and *BRAF* alterations were—as expected—largely mutually exclusive and co‐occurring in only 2.3% of samples. (C) Percentage of *RAS/BRAF*‐altered (SNV or Indel, CN or RE) samples in the rectal cancer cohort. Of all rectal cancer samples (*N* = 9182), 42.1% were *RAS*/*BRAF*‐wt and 57.9% had a known or likely pathogenic *RAS*/*BRAF* alteration. (D) Percentage of *KRAS*, *NRAS*, *HRAS*, or *BRAF* alterations and their pattern of occurrence in the *RAS/BRAF*‐altered rectal cancer cohort (*N* = 5320). *KRAS*, *NRAS*, *HRAS*, and *BRAF* alterations were largely mutually exclusive and co‐occurring in only 2.1% of samples.

In colon cancer, 36.8% of patient samples were *RAS/BRAF*‐wt and 63.2% had a known or likely pathogenic *RAS/BRAF* alteration, respectively (Fig. 1A). Within the *RAS/BRAF*‐altered group, 76.8% harbored a *KRAS* alteration, 5.6% a *NRAS* alteration, 0.1% a *HRAS* alteration, and 15.2% a *BRAF* alteration.

As expected, *RAS*/*BRAF* alterations were co‐occurring rarely in 2.3% of samples (Fig. 1B).

A similar picture was seen in rectal cancer where 42.1% of samples were *RAS/BRAF*‐wt and 57.9% had a known or likely pathogenic *RAS/BRAF* alteration (Fig. 1C). Within the *RAS/BRAF*‐altered group, 82.8% harbored a *KRAS* alteration, 7.4% a *NRAS* alteration, 0.2% a *HRAS* alteration, and 7.5% a *BRAF* alteration. Again, *RAS*/*BRAF* alterations were co‐occurring rarely in 2.1% of samples (Fig. 1D).

**Fig. 2:**
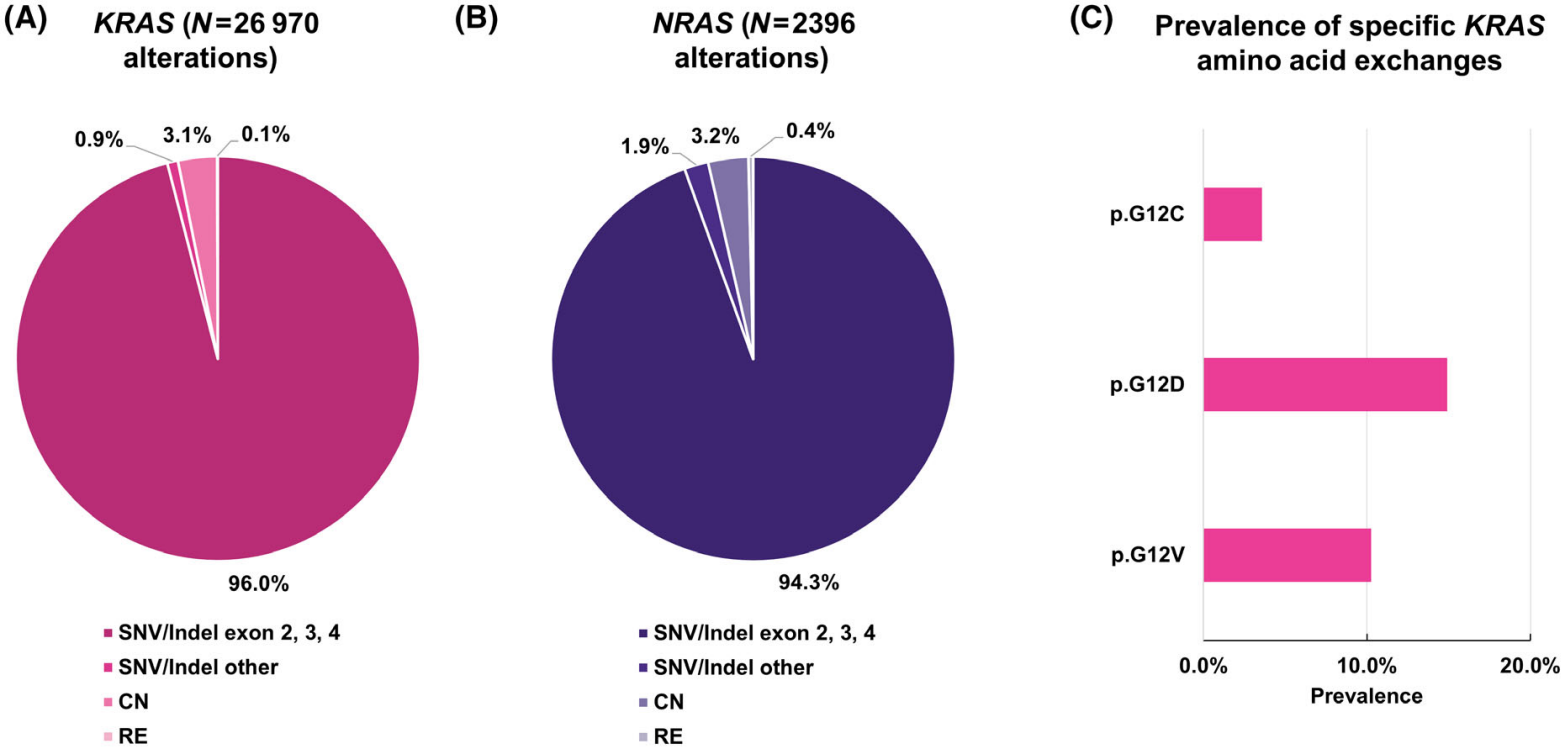
Classification of *KRAS and NRAS* alterations in colorectal cancer (CRC). (A) Percentage of SNVs or Indels in exons 2, 3, and 4, other SNVs or Indels, copy number (CN) alterations and rearrangements (RE) of all *KRAS* alterations (*N* = 26 970), regardless of annotation as pathogenic or presence in a hotspot. 96.0% of all alterations were SNVs or Indels in exons 2, 3, and 4, 0.9% were other SNVs or Indels, 0.1% were RE events, and 3.1% CN events. (B) Percentage of SNVs or Indels in exons 2, 3, and 4, other SNVs or Indels, copy number (CN) alterations and rearrangements (RE) of all *NRAS* alterations (*N* = 2396), regardless of annotation as pathogenic or presence in a hotspot. 94.3% of all alterations were SNVs or Indels in exons 2, 3, and 4, 1.9% were other SNVs or Indels, 0.4% were RE events, and 3.2% CN events. (C) Percentage of samples with the potentially targetable *KRAS* p.G12C, p.G12D, and p.G12V mutations. Relative to all samples (*N* = 51 982) the potentially targetable *KRAS* p.G12C, p.G12D, and p.G12V mutations were present in 3.6%, 14.9%, and 10.3% of samples, respectively.

Of all *KRAS* and *NRAS* alterations in CRC (SNVs and Indels, CN and RE regardless of annotation as pathogenic or presence in a hotspot), SNVs or Indels in exons 2, 3, and 4 constituted 96.0% and 94.3% of all alterations, respectively (Fig. 2A,B) the majority being present in one of the known amino acid hotspots (Fig. S1A,B). In contrast, of all *HRAS*‐SNVs and Indels (*N* = 295), only 48 (16.3%) were classified as likely pathogenic or pathogenic.

Relative to all samples the potentially targetable *KRAS* p.G12C, p.G12D, and p.G12V mutations were present in 3.6%, 14.9%, and 10.3% of samples, respectively (Fig. 2C).

### Agreement between circulating tumor DNA‐based and tissue‐based CGP for detection of *KRAS* , *NRAS*, and *BRAF* V600E mutations

**Fig. 3:**
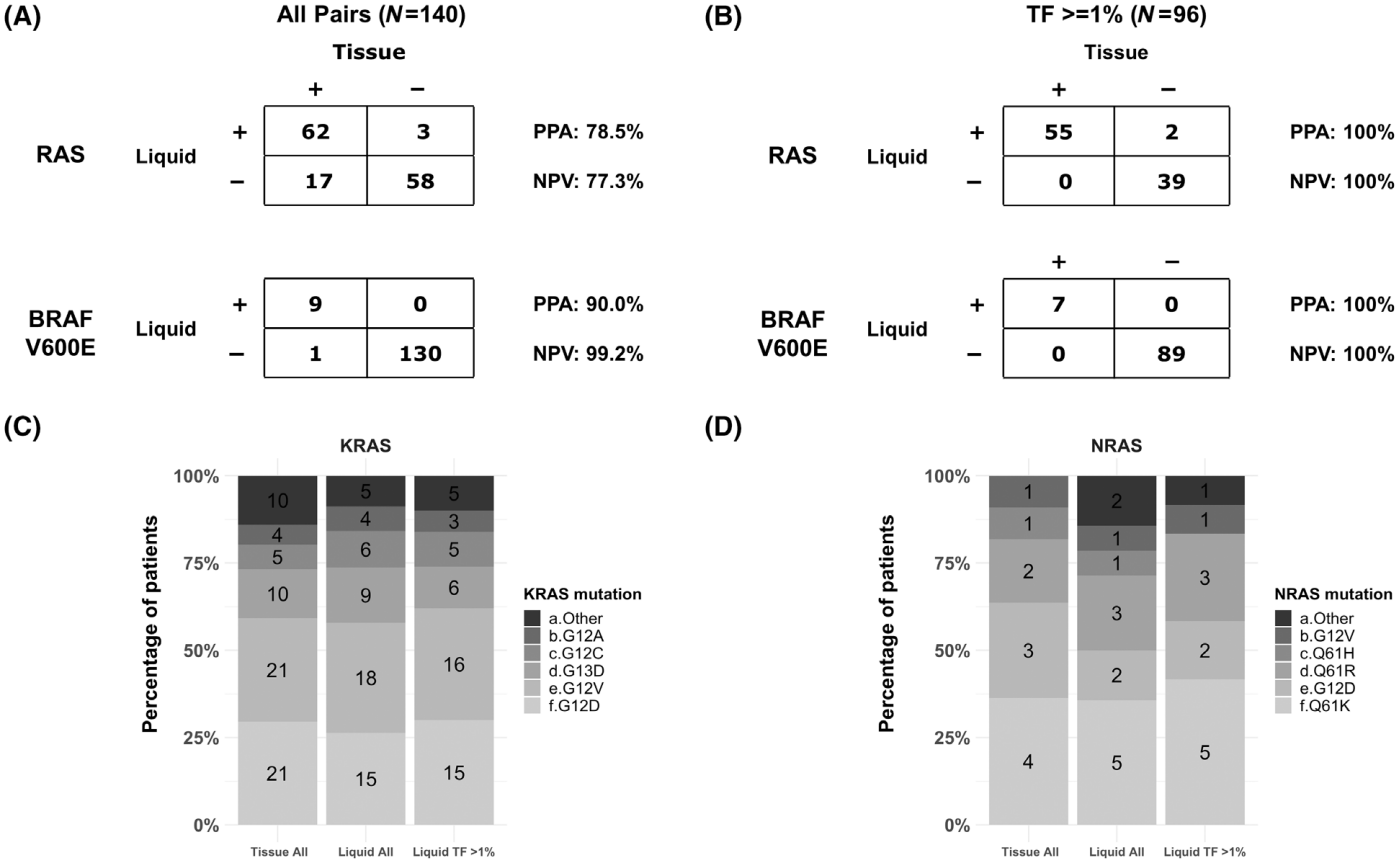
Agreement in detection of *KRAS*, *NRAS*, and *BRAF* V600E mutations between tissue‐ and liquid‐based comprehensive genomic profiling (CGP). (A) Positive percent agreement (PPA) and negative predictive value (NPV) for all pairs (*N* = 140). (B) NPV and PPA for patients with tumor fraction (TF) ≥1% (*N* = 96). (C) *KRAS*‐specific mutations detected in tissue‐based CGP, all liquid‐based CGP, and liquid‐based CGP with TF ≥1%. (D) *NRAS*‐specific mutations detected in tissue‐based CGP, all liquid‐based CGP, and liquid‐based CGP with TF ≥1%.

We next sought to assess agreement in detection of *KRAS*, *NRAS*, and *BRAF* V600E mutations between tissue and liquid‐based (ctDNA) CGP. A subset of the 140 specimens described above were identified to have liquid biopsy‐based CGP results from samples collected within 90 days of the tissue biopsy. Among these 140 pairs, the positive percent agreement (PPA) for *KRAS*, *NRAS*, and *BRAF* V600E mutation detection in liquid was 78.5% and 90.0%, respectively, while the negative predictive value (NPV) was 77.3% and 99.2%, respectively (Fig. 3A).

Ninety‐six of these patients (69%) had a high amount of tumor shed as quantified by a ctDNA tumor fraction (TF) ≥1% in the liquid biopsy. Among these patients, both PPA and NPV for *KRAS*, *NRAS*, and *BRAF* V600E detection in liquid was 100% (Fig. 3B).

The most prevalent *KRAS* alterations were found at similar frequencies among the paired tissue/liquid cohort, including G12D (tissue: 30%; liquid: 26%) and G12V (tissue: 30%; liquid: 32%). The most prevalent *NRAS* mutation detected in tissue and liquid was *NRAS* Q61K (36% in tissue and in liquid whole cohort, and 42% in the subcohort with TF ≥1%) (Fig. 3C,D).

### Frequency of frequently altered genes and immuno‐oncology biomarkers in colon and rectal cancer

In the larger cohort of tissue samples, the genes overlapping between colon and rectal cancer among the top 10 genes with highest alteration frequency were located in relevant signaling pathways, including the *Wnt* pathway (*APC*), *RAS* and *PI3K* pathways (*KRAS*, *PIK3CA*), *TGFB* (*SMAD4*), and *NOTCH* signaling pathway (*SOX9*), in addition to tumor suppressor genes such as *TP53* and *FBXW7* (Fig. S2A,C).

**Fig. 4:**
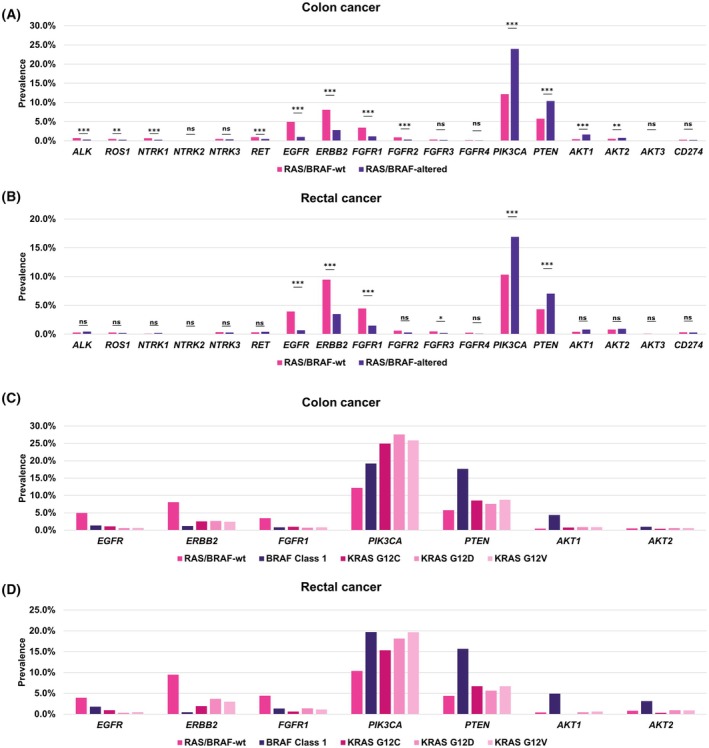
Quantification of potentially targetable alterations by *RAS*/*BRAF* status in colon and rectal cancer. (A, B) Many alterations in genes of the *PI3K* pathway were significantly overrepresented in the *RAS*/*BRAF*‐altered subgroup (*N* = 27 047) in comparison to the *RAS*/*BRAF*‐wt subgroup (*N* = 15 753) (*PIK3CA*, *PTEN*, *AKT1*, *AKT2*), whereas alterations in many RTKs (*NTRK1*, *RET*, *EGFR*, *ERBB2*, *FGFR1*, *FGFR2*) were significantly overrepresented in the *RAS*/*BRAF*‐wt subgroup in colon cancer (A). Similar differences were observed in rectal cancer, where *PIK3CA* and *PTEN* alterations were significantly overrepresented in the *RAS*/*BRAF*‐altered subgroup (*N* = 5320) and *EGFR*, *ERBB2*, *FGFR1*, and *FGFR3* were significantly overrepresented in the *RAS*/*BRAF*‐wt subgroup (*N* = 3862) (B). Statistical comparison was performed with Fisher's exact test, shown are Benjamini–Hochberg adjusted *P*‐values, *P < 0.05, **P < 0.01, ***P < 0.001. (C) Percentage of *RAS*/*BRAF*‐wt (*N* = 15 753), *BRAF* class 1 (*N* = 3474), *KRAS* p.G12C (*N* = 1546), *KRAS* p.G12D (*N* = 6477), or *KRAS* p.G12V‐mutated (*N* = 4360) samples that showed a co‐alteration in one of the seven most commonly altered potentially targetable genes (*EGFR*, *ERBB2*, *FGFR1*, *PIK3CA*, *PTEN*, *AKT1*, *AKT2*) in colon cancer. *BRAF* class 1 mutated samples showed a relatively high prevalence of *PTEN* and *AKT1* alterations. (D) Percentage of *RAS*/*BRAF*‐wt (*N* = 3862), *BRAF* class 1 (*N* = 223), *KRAS* p.G12C (*N* = 313), *KRAS* p.G12D (*N* = 1273), or *KRAS* p.G12V‐mutated (*N* = 971) samples that showed a co‐alteration in one of the seven most commonly altered potentially targetable genes (*EGFR*, *ERBB2*, *FGFR1*, *PIK3CA*, *PTEN*, *AKT1*, *AKT2*) in rectal cancer. *BRAF* class 1 mutated samples showed a relatively high prevalence of *PTEN*, *AKT1*, and *AKT2* alterations.

The five most frequently altered potentially targetable genes in colon and rectal cancer were *PIK3CA* (19.6% and 14.2%), *PTEN* (8.7% and 5.9%), *ERBB2* (4.7% and 6.0%), *EGFR* (2.5% and 2.0%), and *FGFR1* (2.0% and 2.7%). *NTRK* alterations (*NTRK1*/*2*/*3*) were present in 0.9% and 0.5%, *ROS1* alterations in 0.4% and 0.2%, *RET* alterations in 0.6% and 0.4%, and *ALK* alterations in 0.5% and 0.4% of samples in colon and rectal cancer, respectively (Fig. S2B,D). In colon cancer, alterations in the *PI3K* pathway genes *PIK3CA*, *PTEN*, *AKT1*, and *AKT2* were significantly overrepresented in the *RAS*/*BRAF*‐altered subgroup in comparison to the *RAS*/*BRAF*‐wt subgroup (*P* < 0.01, Fisher's exact test with Benjamini–Hochberg correction), only *AKT3* alterations were not significantly overrepresented (*P* > 0.05, Fisher's exact test with Benjamini–Hochberg correction). Alterations of the RTKs *ALK*, *ROS1*, *NTRK1*, *RET*, *EGFR*, *ERBB2*, *FGFR1*, and *FGFR2* were significantly overrepresented in the *RAS*/*BRAF*‐wt group (*P* < 0.01, Fisher's exact test with Benjamini–Hochberg correction). *NTRK2*, *NTRK3*, *FGFR3*, and *FGFR4* did not show a significant overrepresentation (*P* > 0.05, Fisher's exact test with Benjamini–Hochberg correction, Fig. 4A).

A similar pattern was detectable in rectal cancer, where *PIK3CA* and *PTEN* alterations were significantly overrepresented in the *RAS*/*BRAF*‐altered subgroup and *EGFR*, *ERBB2*, *FGFR1*, and *FGFR3* alterations were significantly overrepresented in the *RAS*/*BRAF*‐wt subgroup (Fig. 4B). Further analysis focusing on *BRAF* class 1 as well as *KRAS* p.G12C, p.G12D, and p.G12V and the seven most common potentially druggable alterations revealed that both *PTEN*, *AKT1*, and *AKT2* alterations were enriched in *BRAF* class 1 mutated samples, whereas alteration frequencies of *EGFR*, *ERBB2*, and *FGFR1* were low in all four subgroups in comparison to *RAS*/*BRAF*‐wt samples in colon and rectal cancer (Fig. 4C,D).

The prevalence of immuno‐oncology biomarkers, such as MSI, TMB, and *CD274* (*PD‐L1*), was also explored.

In colon and rectal cancer, 6.1% and 1.7% of samples were MSI‐H, respectively (Fig. S3A,B). 9.6% and 4.6% of samples were TMB‐H (Fig. S4A,B).

Of note, 98.9% of all MSI‐H samples were also TMB‐H by the definition used in this study. In addition, we quantified the prevalence of *POLE* alterations, as germline and somatic *POLE* alterations are a known cause of a high mutational load in CRC [30]. 0.6% and 0.4% of samples were *POLE*‐altered in colon and rectal cancer, respectively (Fig. S2B,D). MSI‐H or TMB‐H samples were significantly more abundant in the *RAS*/*BRAF*‐altered subset in both colon and rectal cancer: In the *RAS*/*BRAF*‐wt subset, 4.5% were MSI‐H and 7.8% TMB‐H, whereas in the *RAS*/*BRAF*‐altered subset 7.1% were MSI‐H and 10.6% TMB‐H in colon cancer (*P* < 0.001, Fisher's exact test, Figs S3C, S4C).

In the *RAS*/*BRAF*‐wt subset, 1.3% were MSI‐H and 4.1% TMB‐H, whereas in the *RAS*/*BRAF*‐altered subset 2.0% were MSI‐H and 5.0% TMB‐H in rectal cancer (*P* < 0.05, Fisher's exact test, Figs S3D, S4D). 0.3% of samples were *CD274*‐altered in both colon and rectal cancer, respectively (Fig. S2B,D). *CD274* was not significantly enriched between *RAS*/*BRAF*‐wt and *RAS*/*BRAF*‐altered samples.

**Fig. 5:**
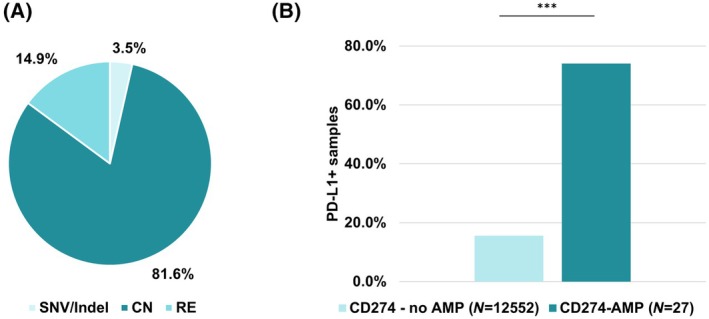
Quantification of *CD274* genomic events and PD‐L1 positivity by immunohistochemistry (IHC). (A) Of all likely pathogenic or pathogenic alterations in *CD274* (*N* = 141), CN events constituted 115 alterations (81.6%), RE events 21 (14.9%), and SNVs or Indels less than 10 alterations (3.5%). All likely pathogenic or pathogenic CN events were amplifications. (B) The subset of samples with a *CD274*‐amplification for which PD‐L1 staining data was available showed a significant enrichment of PD‐L1 positive – low positive (1%–49%) or high positive (≥50%)—samples in the group of samples with amplification (74.1% PD‐L1 positive, *N* = 27) in comparison to samples without amplification (15.6% PD‐L1 positive, *N* = 12 552) (*P* < 0.001, Fisher's exact test).

Most alterations—81.6%—classified as (likely) pathogenic in *CD274* were copy number alterations (Fig. 5A). All likely pathogenic or pathogenic CN events in *CD274* were amplifications. The subset of samples with a *CD274* amplification for which PD‐L1 staining data was available showed a significant enrichment of PD‐L1‐positive samples in the group with amplification (*P* < 0.001, Fisher's exact test, Fig. 5B).

### MSI‐H samples are only more abundant in *BRAF* class 1 and *HRAS‐*mutated CRC samples

**Fig. 6:**
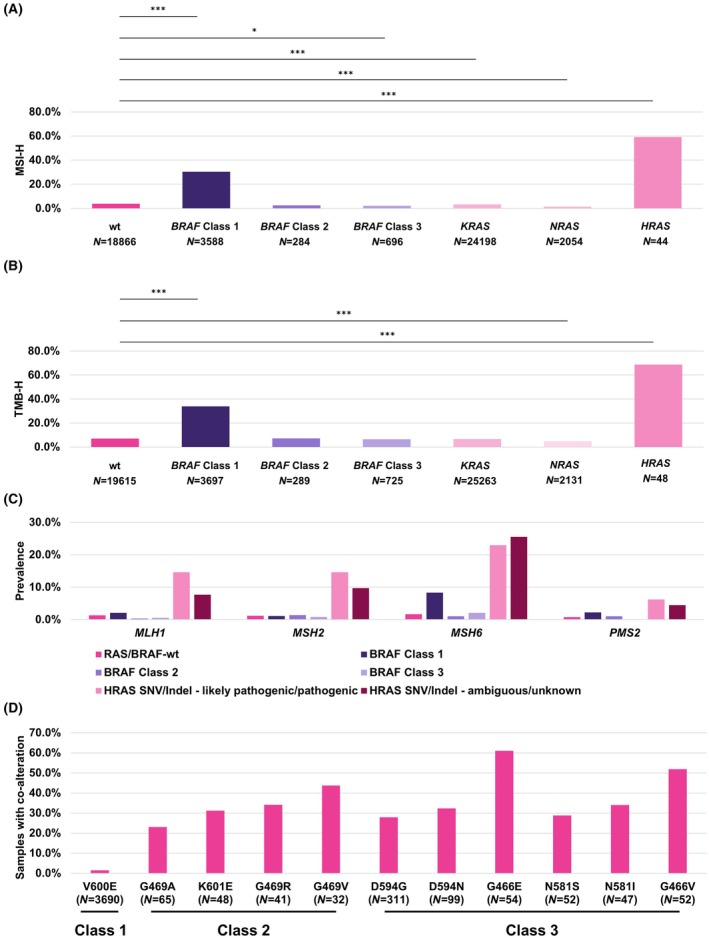
Quantification of microsatellite instability and tumor mutational burden in CRC by *BRAF* mutation class or *RAS* mutation. (A) Microsatellite instability high (MSI‐H) samples were highly and significantly overrepresented in samples that showed a *BRAF* class 1 SNV (*N* = 3588) in comparison to *RAS*/*BRAF*‐wt samples (no likely pathogenic/pathogenic *RAS*/*BRAF* alteration, *N* = 18 866). Samples that showed a *BRAF* class 3 SNV (*N* = 696), or a likely pathogenic/pathogenic SNV or Indel in *NRAS* (*N* = 2054) or *KRAS* (*N* = 24 198) showed a moderate, but significant underrepresentation of MSI‐H samples. The small group of samples with a likely pathogenic/pathogenic SNV or Indel in *HRAS* (*N* = 44) showed the highest proportion of MSI‐H samples. The difference between samples that contained a *BRAF* class 2 SNV (*N* = 284) or Indel and *RAS*/*BRAF*‐wt samples was not significant. Statistical comparison was performed with Fisher's exact test, shown are Benjamini–Hochberg adjusted *P*‐values, *P < 0.05, **P < 0.01, ***P < 0.001. Only significant differences are indicated. (B) Tumor mutational burden high (TMB‐H) samples were highly and significantly overrepresented in samples that showed a *BRAF* class 1 SNV (*N* = 3697) in comparison to *RAS*/*BRAF*‐wt (no likely pathogenic/pathogenic *RAS*/*BRAF* alteration, *N* = 19 615) samples. Samples that showed a likely pathogenic/pathogenic SNV or Indel in *NRAS* (*N* = 2131) showed a moderate, but significant underrepresentation of TMB‐H samples. The difference between samples that contained a *KRAS* (*N* = 25 361), *BRAF* class 2 (*N* = 289), or 3 SNV or Indel (*N* = 725) and *RAS*/*BRAF*‐wt samples was not significant. The small group of samples with a likely pathogenic/pathogenic SNV or Indel in *HRAS* (*N* = 48) showed the highest proportion of TMB‐H samples. Statistical comparison was performed with Fisher's exact test, shown are Benjamini–Hochberg adjusted *P*‐values, *P < 0.05, **P < 0.01, ***P < 0.001. Only significant differences are indicated. (C) Quantification of *MLH1*, *MSH2*, *MSH6*, and *PMS2* alterations by *BRAF*‐mutation class or samples with *HRAS*‐SNV/Indel. *BRAF* class 1 mutated samples (*N* = 3697) showed a higher proportion of alterations in *MLH1*, *MSH6*, and *PMS2*—but not *MSH2*—in comparison to *RAS*/*BRAF*‐wt (*N* = 19 615), *BRAF* class 2 (*N* = 289), or *BRAF* class 3 (*N* = 725) mutated samples, whereas *HRAS*‐mutated samples (likely pathogenic/pathogenic—*N* = 48, unknown/ambiguous—*N* = 247) showed a higher proportion of alterations in all four genes. *MLH1* alterations were present in 2.1% of *BRAF* class 1 mutated samples, 1.3% of *RAS*/*BRAF*‐wt, 0.3% of *BRAF* class 2, 0.6% of *BRAF* class 3 mutated samples 14.6% of samples with a (likely) pathogenic *HRAS* mutation, and 7.7% of samples with a *HRAS* mutation classified as unknown or ambiguous. *MSH2* alterations were present in 1.1% of *BRAF* class 1 mutated samples, in 1.2% of *RAS*/*BRAF*‐wt, 1.4% of *BRAF* class 2, 0.8% of *BRAF* class 3 mutated samples, 14.6% of samples with a (likely) pathogenic *HRAS* mutation, and 9.7% of samples with a *HRAS* mutation classified as unknown or ambiguous. *MSH6* alterations were present in 8.3% of *BRAF* class 1 mutated samples, in 1.7% of *RAS*/*BRAF*‐wt, 1.0% of *BRAF* class 2, 2.1% of *BRAF* class 3 mutated samples, 22.9% of samples with a (likely) pathogenic *HRAS* mutation, and 25.5% of samples with a *HRAS* mutation classified as unknown or ambiguous. *PMS2* alterations were present in 2.2% of *BRAF* class 1 mutated samples, in 0.8% of *RAS*/*BRAF*‐wt, 1.0% of *BRAF* class 2, 0.1% of *BRAF* class 3 mutated samples, 6.3% of samples with a (likely) pathogenic *HRAS* mutation, and 4.5% of samples with a *HRAS* mutation classified as unknown or ambiguous. (D) Characterization of *RAS* co‐alterations with *BRAF* mutations based on the 10 most common *BRAF*‐SNVs in CRC. Whereas *RAS* co‐alterations were rare in samples with *BRAF* V600E mutation (class 1, *N* = 3690), both samples with *BRAF* class 2 (G469A—*N* = 65, K601E—*N* = 48, G469R—*N* = 41, G469V—*N* = 32) mutations as well as class 3 mutations (D594G—*N* = 311, D594N—*N* = 99, G466E—*N* = 54, N581S—*N* = 52, N581I—*N* = 47, G466V—*N* = 52) frequently co‐occurred with *RAS*‐SNVs or Indels (restricted to likely pathogenic or pathogenic mutations).

Importantly, this effect was driven by *BRAF* class 1 mutated samples that showed a significant overrepresentation (adjusted *P* < 0.001, Fisher's exact test with Benjamini–Hochberg correction) of MSI‐H samples in comparison to *RAS*/*BRAF*‐wt samples in all CRC cases. Among the *BRAF* class 1 mutated samples, the percentage of MSI‐H samples was 30.3%, whereas only 3.9% of *RAS*/*BRAF*‐wt samples were MSI‐H. In addition, samples with a (likely) pathogenic *HRAS*‐SNV or Indel showed a significant overrepresentation of MSI‐H samples (adjusted *P* < 0.001, Fisher's exact test with Benjamini–Hochberg correction), the percentage of MSI‐H samples was 59.1%. *BRAF* class 3 (2.2% MSI‐H), *NRAS* (1.5% MSI‐H), and *KRAS* (3.2% MSI‐H) mutated samples showed an underrepresentation of MSI‐H samples. There was no significant difference between *RAS*/*BRAF*‐wt and *BRAF* class 2 (2.5% MSI‐H) mutated samples (*P* > 0.05, Fisher's exact test with Benjamini–Hochberg correction, Fig. 6A). In line with this finding, *BRAF* class 1 mutated samples showed a higher proportion of alterations in *MLH1*, *MSH6*, and *PMS2*—but not *MSH2*—in comparison to *RAS*/*BRAF*‐wt, *BRAF* class 2, or *BRAF* class 3 mutated samples. Samples with a *HRAS*‐SNV or Indel showed an even higher proportion of alterations in *MLH1*, *MSH6*, *PMS2*, and *MSH2*—regardless of the classification of the *HRAS* variant as likely pathogenic/pathogenic or ambiguous/unknown (Fig. 6C). The results for TMB were similar, with the exception that neither *BRAF* class 2 (7.3% TMB‐H) nor 3 (6.5% TMB‐H) mutated samples showed a significant difference in comparison to *RAS*/*BRAF*‐wt (7.1% TMB‐H) samples (*P* > 0.05, Fisher's exact test with Benjamini–Hochberg correction). Among the *BRAF* class 1 mutated samples, the percentage of TMB‐H samples was 33.9%, in the samples with *HRAS* mutation 68.8% and in *KRAS*‐ or *NRAS*‐altered samples 6.7% and 4.8% percent, respectively (Fig. 6B).

### Both *BRAF* class 2 and 3 mutations co‐occur frequently with *RAS* ‐SNVs or Indels in CRC

Finally, we quantified how many samples show *BRAF* class 1, 2, or 3 mutations and a co‐occurring *RAS*‐SNV or Indel, based on the 10 most common *BRAF* mutations (with information regarding their class assignment available) in our cohort. Interestingly, while *BRAF* V600E co‐occurred with *RAS*‐SNVs or Indels rarely (1.4% of samples), *BRAF* class 2 (G469A—23.1%, K601E—31.3%, G469R—34.1%, G469V—43.8%) mutations as well as class 3 mutations (D594G—28.0%, D594N—32.3%, G466E—61.1%, N581S—28.8%, N581I—34.0%, G466V—51.9%) frequently co‐occurred with *RAS*‐SNVs or Indels (restricted to likely pathogenic or pathogenic) (Fig. 6D).

## Discussion

Real‐world data (RWD)‐based clinical genomic profiling has provided important insights for precision medicine due to the ability to access data from large cohort sizes, *for example*, for mutations which were not sufficiently represented in clinical trials or which are rare in general [15, 16, 31, 32].

In this study, RWD confirmed that the vast majority of *RAS* alterations in CRC are SNVs or Indels in exons 2, 3, and 4 and occur in the known hotspots by both tissue and liquid‐based CGP in *KRAS* and *NRAS*. Agreement between tissue‐based and liquid‐based CGP for detection of *RAS* and *BRAF* alterations results in PPA and NPV of 100% when liquid biopsy specimens containing ctDNA TF ≥1% were selected. The concentration of ctDNA itself is known to depend on tumor stage as well as biological differences in ctDNA release between tumors [33, 34].

We observed, in accordance with earlier observations, enrichment of RTK alterations in the *RAS*/*BRAF*‐wt and of *PI3K* pathway alterations in the *RAS*/*BRAF*‐altered subgroup [10, 11, 35, 36]. *PTEN*, *AKT1*, and *AKT2* alterations showed a high prevalence in *BRAF* class 1 mutated samples.

Consistent with previous studies in metastatic CRC [21], both TMB‐H and MSI‐H samples were significantly more abundant in the *RAS/BRAF*‐altered cohort, an effect that is driven by *BRAF* class 1 mutated samples. This confirms co‐targeting of *BRAF* class 1 mutants with both AKT inhibition and/or immune checkpoint inhibition as attractive therapeutic concept in this subgroup. The optimal treatment sequence for this subgroup will require additional precision medicine trials focusing on the treatment sequence and response toward immune checkpoint inhibition and/or AKT inhibition. Our large cohort size enabled us to characterize *BRAF* class 2 and 3 mutated samples as well as *HRAS*‐mutated samples separately. Samples with *BRAF* class 2 and class 3 mutation did not show any enrichment of MSI‐H samples in agreement with earlier reports [21, 37]. Interestingly, the very small group of samples with *HRAS*‐SNVs or Indels showed a distinct behavior with a strong enrichment in MSI‐H samples and a co‐alteration pattern of genes involved in mismatch repair.

While the frequencies of concomitant *RAS* mutations are similar to earlier studies in non‐*BRAF* V600E‐mutated CRC [38], we found that *KRAS* and *NRAS* co‐alterations were frequent in both *BRAF* class 2 and 3 mutated samples, and not confined to class 3 mutated samples [3, 37]. A reason for this may be the treatment history of the patients, as, *for example*, treatment with anti‐EGFR antibodies is known to enrich *MAPK* pathway alterations [39].

The strength of our study is the large cohort size (*N* = 51 982) that enables generation of meaningful data from an unprecedented number of cases with rare alterations. In summary, RWD mining from both tissue and liquid biopsy‐based CGP can provide valuable insights and has the potential to play an important role in informing key clinical decision‐making such as comprehensive clinical study design including patient selection and stratification criteria.

## Conflict of interest

MS and JS are employees of Merck Healthcare KGaA, Darmstadt, Germany. XW and ZF are employees of EMD Serono Research and Development Institute, Inc., Billerica, MA, USA, an affiliate of Merck KGaA, Darmstadt, Germany. JH is an employee of Merck Serono Middle East FZ‐Ltd., Dubai, United Arab Emirates, an affiliate of Merck KGaA, Darmstadt, Germany. JCFQ and LWP are employees of Foundation Medicine Inc, Cambridge, MA, USA, a wholly owned subsidiary of Roche, and have equity interest in Roche. JFH was employed by Foundation Medicine and owned stock in Roche Holding AG while contributing to the manuscript.

## Author contributions

ZF and JS: Conception and design. MS, LWP, and JCFQ: Analysis and interpretation of data. MS, XW, JH, JCFQ, LWP, JFH, JS, and ZF: Writing, review, and/or revision of the manuscript. JFH: Administrative, technical, or material support. ZF: Study supervision.

## Supporting information


**Fig. S1.** Overview of *KRAS* and *NRAS* mutations by hotspot in CRC.

**Fig. S2.** Prevalence of common and potentially targetable alterations in colon and rectal cancer.

**Fig. S3.** Quantification of microsatellite‐instability in colon and rectal cancer by *RAS*/*BRAF*‐status.

**Fig. S4.** Quantification of tumor mutational burden in colon and rectal cancer by *RAS*/*BRAF*‐status.

## Data Availability

Data used for the analysis of tissue alterations have been retrieved via the commercially available FoundationInsights web platform. Only the final results as presented in this manuscript are available. Patient‐level liquid biopsy data are proprietary to Foundation Medicine, Inc.
