# Supplementary figures and images for: Real‐world genomic landscape of colon and rectal cancer
Source: FEBS Open Bio. 2025 Jan 26;15(4):674–85. doi: 10.1002/2211-5463.13957 (PMC11961397; doi:10.1002/2211-5463.13957)

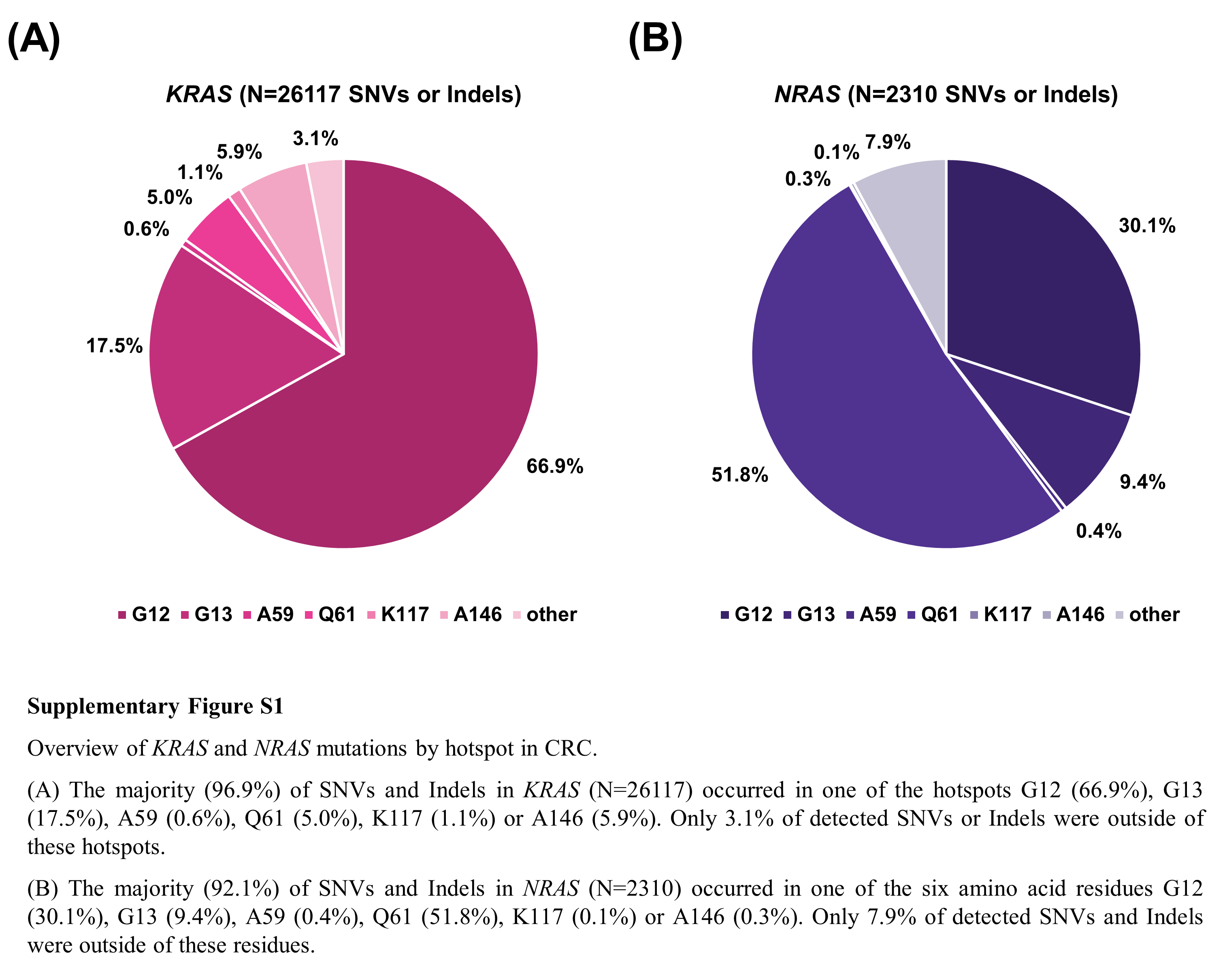

Supplement: Supplementary file 1 — Fig. S1. Overview of KRAS and NRAS mutations by hotspot in CRC. [file FEB4-15-674-s001.tif]

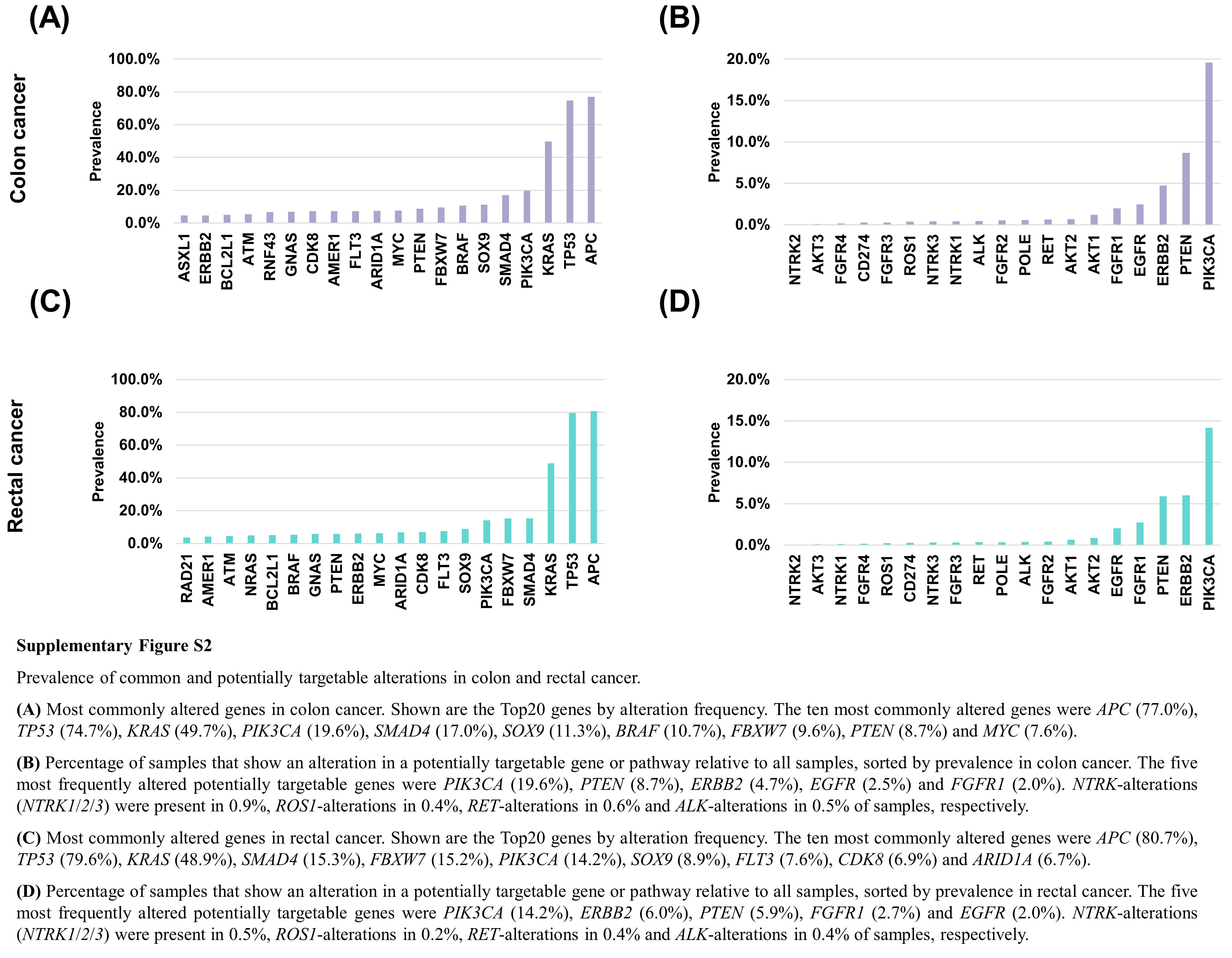

Supplement: Supplementary file 2 — Fig. S2. Prevalence of common and potentially targetable alterations in colon and rectal cancer. [file FEB4-15-674-s004.tif]

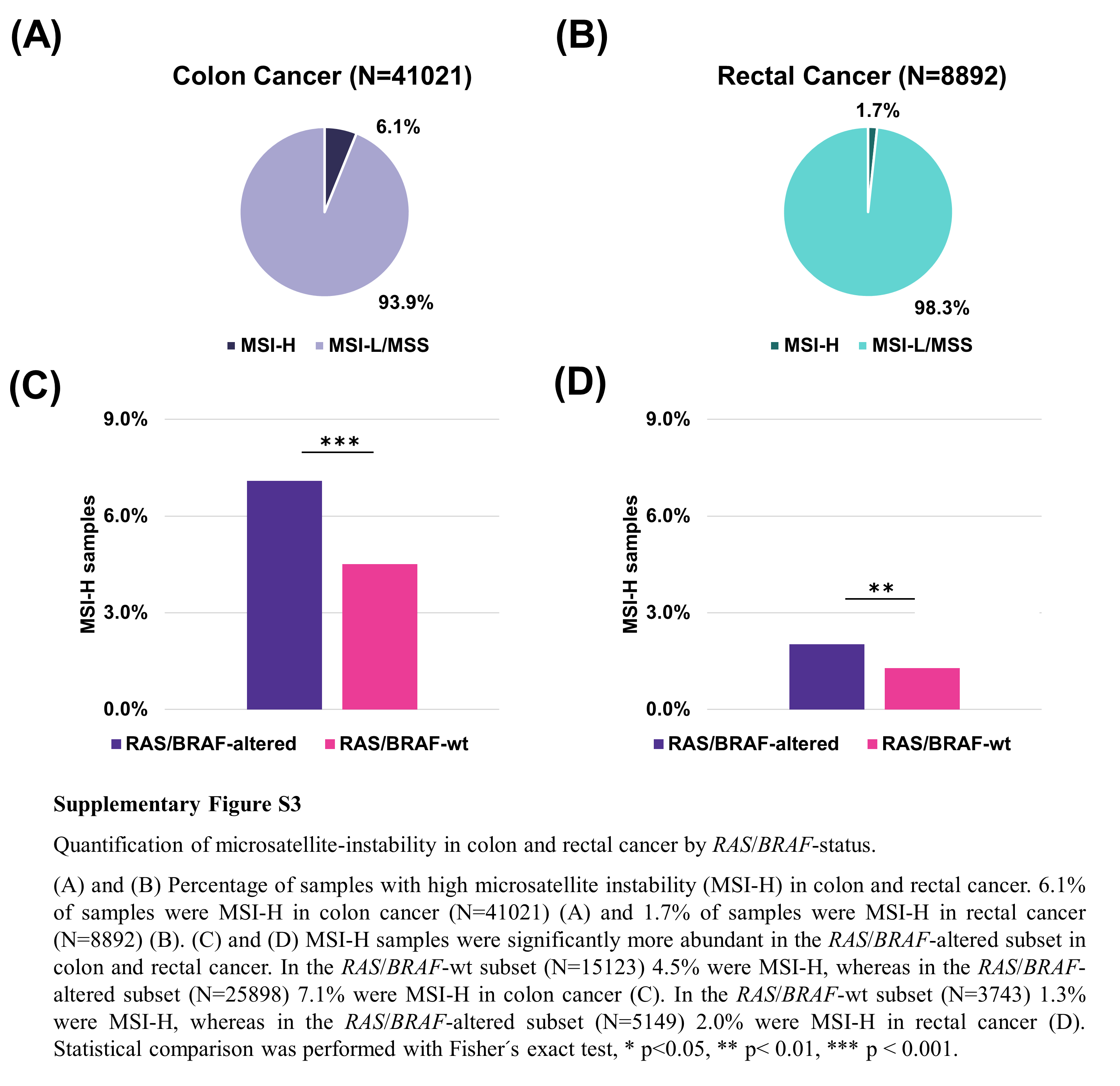

Supplement: Supplementary file 3 — Fig. S3. Quantification of microsatellite‐instability in colon and rectal cancer by RAS/BRAF‐status. [file FEB4-15-674-s002.tif]

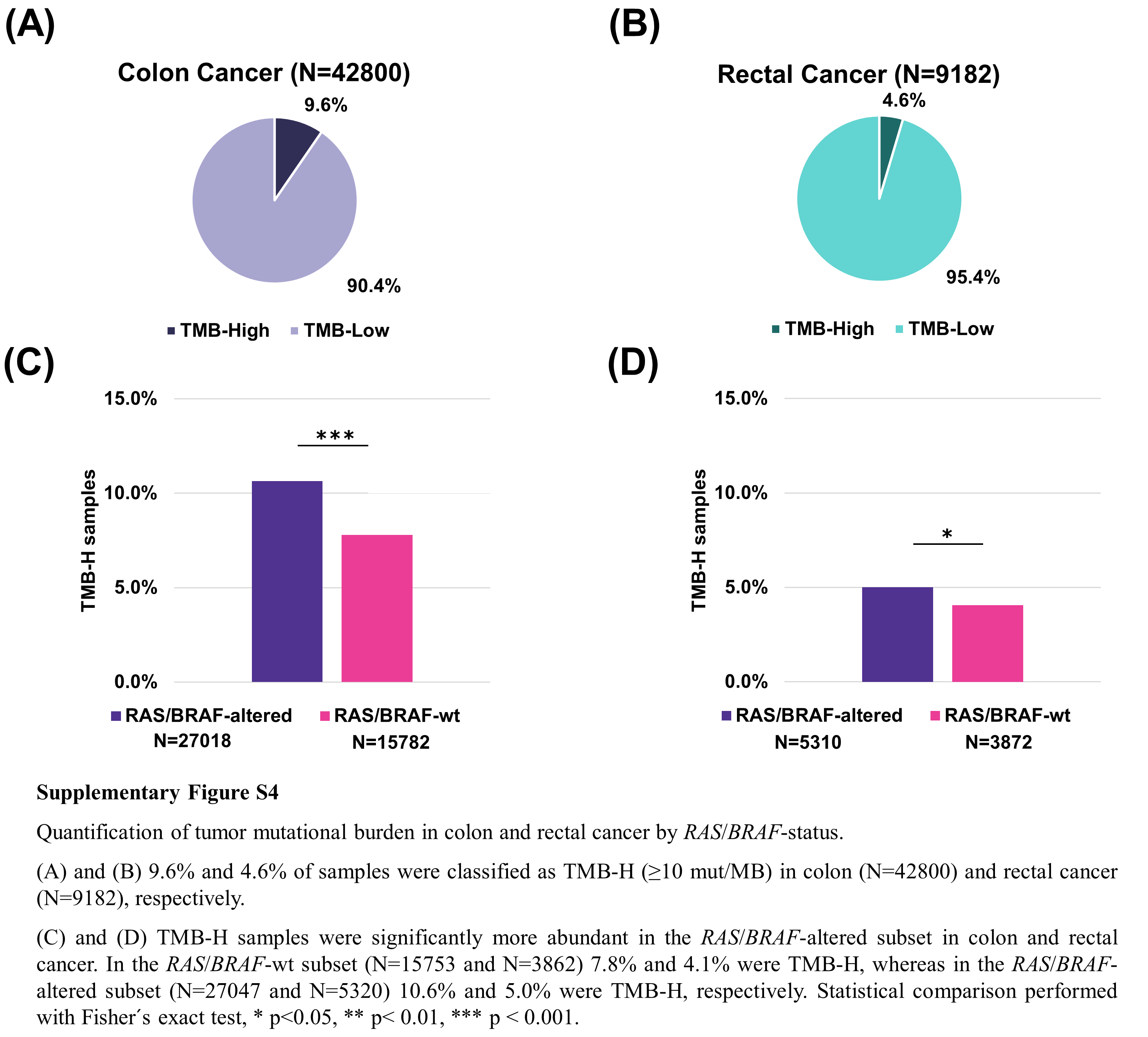

Supplement: Supplementary file 4 — Fig. S4. Quantification of tumor mutational burden in colon and rectal cancer by RAS/BRAF‐status. [file FEB4-15-674-s003.tif]
